# Combining social protection interventions for better food security: Evidence from female-headed households in Amhara region, Ethiopia

**DOI:** 10.1371/journal.pone.0283812

**Published:** 2024-02-26

**Authors:** Essa Chanie Mussa, Dessie Agegnehu, Emmanuel Nshakira-Rukundo

**Affiliations:** 1 Department of Agricultural Economics, University of Gondar, Gondar, Ethiopia; 2 Ebinat Woreda Agriculture and Livestock Office, Ebinat, Amhara Region, Ethiopia; 3 RWI-Leibniz Institute for Economic Research, Essen, Germany; 4 The German Institute of Development and Sustainability (IDOS), Bonn, Germany; 5 Institute for Food and Resource Economics, the University of Bonn, Bonn, Germany; 6 Apata Insights, Kampala, Uganda; University of Nairobi, KENYA

## Abstract

Ethiopia introduced its flagship poverty-targeted social protection program, the Productive safety net program (PSNP), in 2005 and Community-Based Health Insurance (CBHI) in 2011. Although both programs operate in several districts with some overlaps, evidence is scarce on how these large-scale programs jointly affect the food security of vulnerable groups. This study examines the impacts of a combination of these programs on food security outcomes among female-headed households in a chronically food-insecure and drought-prone district. Cross-sectional data were collected from 365 female-headed households selected through multi-stage sampling technique and analyzed using Inverse-probability-weighted regression adjustment (IPWRA) strategy to assess the effect of the programs on food security. The results show that while 63.6% of sample households are enrolled in CBHI and 48.8% are beneficiaries of PSNP’s conditional cash transfer (CCT) component, membership in both social protection programs was 38.9%. The IPWRA analysis finds that inclusion in the CCT combined with CBHI, on average, increased dietary diversity score by 0.918 (95% CI 0.779–1.057) and food consumption score by 0.576 (95% CI 0.464–0.688). It also reduced household food insecurity access scale by 8.658 (95% CI -9.775 – -7.541). In all assessments, a combination of CBHI and CCT always produced results of a larger magnitude than each of CBHI and CCT alone. The findings provide evidence of the potentials of integrating social protection programs to increase food security outcomes among the most vulnerable and marginalized groups in a developing country. In addition, the results have also useful implications to achieve sustainable development goals related to ending hunger and achieving food security among vulnerable groups.

## 1. Introduction

Food security exists when “all people, at all times, have physical and economic access to sufficient, safe and nutritious food to meet their dietary needs and food preferences for an active and healthy life” [1]. This suggests that food insecurity is the absence of one or more of these conditions (i.e. availability, access, utilization and stability) [2]. Globally, the proportion of people defined as chronically undernourished, i.e. people who are unable to acquire enough food to meet daily minimum dietary energy requirements declined from 18.6% in 1990–2002 to 11% in 2014–16 [3]. In sub-Saharan Africa, the proportion of the chronically undernourished population declined from 33% during 1990–2002 to 19% in 2014–16 [4]. However, after such a prolonged decline, global hunger has increased in recent years due mainly to several reasons including climate change, conflict [5], and more recently, the COVID-19 pandemic [6]. In 2019, about 750 million people (one in every nine people globally) were severely food insecure and approximately 2 billion people were moderately or severely food insecure (did not have regular access to safe, nutritious, and sufficient food) [7]. In addition, following the outbreak of the COVID-19 pandemic, the number of people who faced hunger and food insecurity increased globally [8]. The 2022 world food security and nutrition report showed that 828 million people globally faced hunger and 2.3 billion people were moderately or severely food insecure in 2021 [8]. Moreover, the number of undernourished people in Africa is growing faster than in any other region of the world [7]. The prevalence of moderate and severe food insecurity in Ethiopia has also increased in the past few years. The proportion of the population with severe or moderate food insecurity has increased from 56.7 million people in 2014–16 to 63.2 million people in 2018–20 [7], a net increase of about 7 million people.

Moreover, women are more likely to be food insecure than men [7], and female-headed households are more likely to experience food insecurity compared to their male-headed counterparts [9–14]. For example, a review of 15 studies by Negesse et al. [12] in Ethiopia shows that food insecurity among female-headed households was 66% and they are almost twice more likely to be food insecure compared with male-headed households. Various factors may contribute to this condition, including differences in asset ownership [10] such as agricultural land [3]. What is more, female-headed food insecure households are also more likely to adopt consumption-based coping mechanisms compared to their male-headed counterparts [9].

One of the avenues for reducing food insecurity in low-income countries has been the expansion of social protection interventions. In this regard, the government of Ethiopia introduced the rural PSNP in 2005, as part of its food security program, to fundamentally address the problems of food insecurity. The PSNP aims to reduce vulnerability to food insecurity through the provision of temporary employment opportunities for households with able-bodied members for cash and/ or in-kind payments and unconditional cash transfers to labor-constrained households [15]. However, existing literature shows mixed impacts of the PSNP on food security outcomes. Some studies find that PSNP improves various food insecurity indicators such as consumption expenditure [16], caloric acquisition [17], and the number of months households were food secure [18]. However, other studies have, on the contrary, found no improvements in household food security and nutrition indicators due to PSNP [19, 20]. There is also limited evidence on the impact of PSNP when the program is integrated with other large-scale social protection programs such as CBHI. One exception examined the joint role of the PSNP and transfers from other food security programs (OFSP) and found that public works (PW), the CCT component of the PSNP, and OFSP beneficiary households are more likely to be food secure [17].

To increase the comprehensiveness of social protection, more recently, countries have tended towards combining instruments, leveraging on individual contributions to reduce the limitations of each instrument. Against this backdrop, this study aims to investigate the impacts of two social protection programs in Ethiopia, the CCT component of the PSNP and the CBHI, on food security outcomes of female-headed households in Ebinat district, Amhara region, Ethiopia. The PSNP currently covers close to 8 million people with either the public works (PW) intervention or the unconditional CT intervention to labor-constrained households including elderly, orphaned children, and people with disabilities [21]. The PSNP identifies female-headed households as one of the most vulnerable groups, hence gender-sensitive elements have been incorporated into the program. Female-headed households with labor shortages receive household-level support on their land preparation and cultivation, and are also prioritized in the livelihoods and cash transfer components of the program [21]. This enables beneficiary households to engage in on-farm and off-farm income-generating activities and thus build resilience [22].

Alongside the PSNP, the government of Ethiopia also started a pilot community-based health insurance (CBHI) in 13 rural woredas across the country in 2011. The program, through a voluntary and risk-pooling mechanism, aims to provide a risk protection system to households in the rural and informal sectors [23]. The CBHI benefit package covers all outpatient and inpatient services at the contracted public health centers and hospitals (through referral system) except dental, optic and cosmetic procedures [24]. Most recent enrollment estimations indicate that about 45–50% of the population is enrolled [25, 26]. In 2018, overall CBHI enrolment in the study woreda was 64%. Furthermore, annual premiums are calculated based on household size: households with up to five members pay ETB 240 (8.7 USD, based on the average exchange rate in 2018); households with six and seven members pay ETB 290 (10.5 USD), and those with eight members or more pay ETB 340 (12.3 USD) [27].

This study, therefore, aims to assess the effect of belonging in one or both of these social protection interventions on food security in female-headed households. In this way, the study contributes to the very few studies that assess the effects of combined social protection interventions in low-income countries. Moreover, none of the existing studies assess extremely vulnerable people such as female-led households. This study is therefore not only contributory to this meager literature but also formative in studying a largely understudied sub-population. In addition, this study also provides useful evidence on ways to ensure the effectiveness of development programs to address food insecurity among the most vulnerable households covered by large-scale development programs.

The results of this study are important for policy in two dimensions. First, they highlight the importance of precise targeting and retargeting of extremely vulnerable households such as poor female-headed households. By focusing on this sub-group of the population, the study highlights not only on their vulnerability but also on the impacts that accrue with participation in these social protection interventions such as cash transfers and health insurance. Secondly, the study provides additional evidence on the usefulness of complimentary development interventions. Conventionally, governments and development organizations have looked at individual interventions separately and have not considered the benefits of combining interventions for the most vulnerable groups. More recently, evidence has emerged on combined interventions including that from Ethiopia [28–30] and Ghana [31, 32], where health insurance and cash transfer interventions have been targeted to same households. None of the studies assess the specific situation of female-headed households, who are more likely to have more multiple vulnerabilities than other average households. This study fills this gap.

## 2. Literature review

To some extent, policy choices between one program (e.g. cash transfers) and another program (e.g. health insurance) find no significant differences [33], and therefore the interest in combining these interventions is increasing. Several studies assess the complementarities between various social protection interventions. For instance, participation in cash transfer programs increases enrolment in health insurance programs [28, 30, 31, 34] and increased utilization of health services [29].

One study by De Groot et al. [32] looks at the effect of combined programs (Ghanaian cash transfer program paired with health insurance fee exemption) on food security in Ghana. The study finds no significant effects among participating households on nutrition outcomes. However, although household-level food security increased, child meal frequency reduced, suggesting a possible intrahousehold allocation of resources to adults.

Several studies assess the effects on various economic and social outcomes including labor supply, employment, and intimate partner violence. Osei and Lambon-Quayefio [35] used the Ghana interventions to study labor supply and find that the program increased the probability of transitioning from unemployment to employment, increasing wage employment among both males and females but also reduced the probability of transitioning to self-employment among young people. Similarly, Peterman et al. [36] study the effect of LEAP 1000 on intimate partner violence and find some significant effects on emotional, physical, and combined intimate partner violence. The authors also find reduction in household poverty, increases in household expenditure, and women’s empowerment in form of savings. Barrington et al. [37] also qualitatively assess the interaction between health insurance and cash transfers finding that poverty was the main determinant of intimate partner violence and that cash transfers were not a panacea for gender norms and the role that maintain women’s economic insecurity. One study in Ethiopia also finds that combining the Ethiopian PSNP and community-based health insurance led to lower household debt and higher labor market participation [29].

Literature assessing combined social protection interventions is growing but still very thin. Only a few studies assess the situation of extremely vulnerable sub-groups such as women [36] or women-headed households [30]. Only one study [32] assesses the combined effects of the Ghanaian cash transfer program and health insurance fee exemption on food security. This paper, therefore, attempts to fill this gap by investigating the effect of insurance, cash transfers, and the combination of the two programs.

Our study, in this respect, is the first attempt that tries to evaluate the joint impacts of receiving conditional CT through the PSNP’s PW component and enrolment in CBHI on household food security outcomes. In addition, we also provide evidence on the impacts of enrolment in CBHI and membership in CCT separately on food security outcomes. Unlike the past studies in Ethiopia that consider the general population or PSNP-participating households alone, we take the gender dimension and consider rural female-headed PSNP-participating and non-participating households. Female-headed households are identified as one of the most vulnerable and disadvantaged groups in Ethiopia [15]. Furthermore, our sample PSNP-participating households are drawn from the PW component of the PSNP.

## 3. Methods

### 3.1 Study area, sampling, and data

The study is conducted in Ebinat *woreda* (district), one of the chronically food-insecure and drought-prone districts of Amhara region. The PSNP was introduced in the district in 2005 by targeting 14,767 male-headed and 7,029 female-headed food-insecure households. Ten years later, in 2015, the district also launched CBHI to protect households in the rural and informal sectors against health-related risks such as high out-of-pocket health expenditure [38]. As of 2018, the CBHI enrolment in the district among eligible households was 64% [27]. In 2020, the district had 22,518 rural households (14,690 male-headed and 7,828 female-headed) of which the number of PW participating households was 6,339 (3,299 male-headed and 3,040 female-headed). Furthermore, 884 households (604 male-headed and 280 female-headed) were graduated from PSNP in 2020 [38].

Ebinat district was purposively selected due to the presence of PSNP and CBHI programs. We used a multi-stage sampling technique to select 365 female-headed households (178 CCT participants and 187 non-participants) from 3 rural villages/ communities. While treatment households were selected randomly from the village (K*ebele*) beneficiary list, non-participant households were selected from the list of female-headed households that were shortlisted for targeting by the Kebele food security task forces (KFSTF) in the two years before the study (retargeting rounds) but were excluded due to budget constraint. These households have never been in the PSNP. In the past five years, between 51.9% and 55.7% of food-insecure female-headed households were excluded from being targeted by the PW due to budget constraints [38]. During February 9–15, 2021, household listing was conducted in villages where there were no or incomplete administrative records to select comparison households. The sample size was calculated using Cochran’s formula [39] using a 5% margin of error, 95% confidence level (z = 1.96), and proportion of p = 0.39 (the ratio between the number of female-headed households in the PW component and total number of female-headed households in the district in 2020). To account for potential non-response, we increased the sample size for beneficiary households by 10% and decreased the comparison sample by 10%.

We collected cross-sectional data by interviewing female heads during March 5–28, 2021. Trained enumerators on the survey questionnaire and research ethics conducted the data collection. Survey instruments were translated into the local language (Amharic) and all interviews were conducted in Amharic.

### 3.2 Ethics and consent to participate

The study has been approved by the Department Research Committee (DRC) at the department of Agricultural Economics, University of Gondar, Ethiopia, for its compliance with institutional procedures and ethics requirements. Oral consent has been obtained from all survey respondents during interviews to participate in the study and consent was obtained from them to use their anonymized information. All methods were performed following the relevant guidelines and regulations.

### 3.3 Variable measurements

#### 3.3.1 Treatment indicators

Households may have one of the following four participation statuses with respect to the two social protection programs: 1) being members of both CCT and CBHI programs, 2) participation in CCT only, 3) CBHI enrolment only, and 4) neither of the two. Accordingly, households are coded 1 (treatment) if they are members of both CCT and CBHI programs and 0 (comparison) if they are members of one of the programs only or none to evaluate the impacts of being members of the two programs. Furthermore, to measure the impact of participation in CCT component of the PSNP, households are coded 1 (treatment) if they are beneficiaries of CCT component and 0 (comparison) if they are non-CCT beneficiaries. Finally, in order to measure the impacts of CBHI enrollment on food security outcomes, households who are members of CBHI are coded 1 (treatment), while non-insured households are coded 0 (comparison).

#### 3.3.2 Food security indicators: Purpose and measurement

Considering the multidimensionality of food insecurity, this study used the three most commonly used food security measurements at the household level: dietary diversity score (HDDS), food consumption score (HFCS), and food insecurity access scale (HFIAS) to capture different aspects of the food (in)security among female-headed households. The key reason for using three measures was the necessity to capture the different dimensions [40, 41]. For instance, Maxwell and colleagues [41] compared different measures, including the food consumption score, coping strategies index, the household diet diversity score, the reduced coping strategies index, the household food insecurity access scale, the household hunger index, and a self-assessed measure of food insecurity in the Tigray Region. They concluded that each of these measures captured different dimensions due to the underlying aspects of food insecurity they measured. They also observed that each indicator was likely to be sensitive in measuring certain aspects of severity. Moreover, they also observed that the choice of cut offs matters for the measurement of food insecurity. One key recommendation then was to use multiple measurements to capture multiple dimensions especially since there was no “gold standard measure” of food insecurity.

*Household Dietary Diversity Score (HDDS)*: The HDDS is one of the commonly used food security measurements with some desirable qualities. Firstly, food group consumption data required to measure dietary diversity is easy to collect [2]. Secondly, it is also shown that dietary diversity has positive associations with nutrient quality of individual’s diets and serves as a proxy for the access dimension of food security [42]. The indicator also demonstrates positive associations with socioeconomic conditions of households such as income [42]. Thirdly, the indicator also uses a 24-hour recall period which is easier to recall compared to other reference periods such as last seven days or four weeks.

Following Swindale and Bilinsky [42], we used the following 12 food groups to calculate the HDDS: Cereals; roots and tubers; vegetables; fruits; meat and poultry; eggs; fish and seafood; pulses, legumes and nuts; milk and milk products; fats and oils; sweets and honey; and miscellaneous such as spices. Respondents were asked if any member in the household consumed the specific food group during the previous 24 hours. Accordingly, the “yes” responses were coded as 1, otherwise coded as 0. The HDDS for each household was calculated by summing responses for the 12 food groups, resulting in scores ranging from 0 to 12. Due to lack of standard cutoffs for defining food insecurity using HDDS [2], the household-level scores were used for the analysis in this study. Higher numbers show more (diversified) food groups consumed in the 24-hour period.

*Household Food Consumption Score (HFCS)*: The HFCS combines data on dietary diversity, frequency of food groups consumed using 7-day recall period, and weights, showing the relative nutritional value of the food groups [43]. The HFCS is validated based on data from low-income countries and demonstrates positive association with calorie consumption per capita [44]. Accordingly, following the World Food Programme [45], data were collected on 16 food items and these were aggregated into 9 food groups during analyses [45, 46]. These food groups include main staples, pulses, vegetables, fruit, meat and fish, milk, sugar, oil and condiments [45]. The consumption frequency of each food group in the past seven days is then multiplied by assigned weights (i.e. 2, 3, 1, 1, 4, 4, 0.5, 0.5 and 0 for main staples, pulses, vegetables, fruit, meat and fish, milk, sugar, oil and condiments, respectively). However, due to extremely low HFCS among our sample (Table 3), on average categorized as “poor” based on WFP classification, this study used the scores as a continuous variable. We used the HFCS to measure household-level caloric intake and quality of diet.

*Household Food Insecurity Access Scale (HFIAS)*: The HFIAS is an experience-based food insecurity indicator, capturing the access dimension of food security based on households’ behavioral and psychological manifestations of insecure food access [2, 47]. It uses the past 4 weeks (30 days) as a reference period [48]. The indicator is used to assess prevalence of household food insecurity of a population. Accordingly, first, respondent is asked an “occurrence question” if the household experienced the specific condition in the past 4 weeks, and, if yes, it is followed by “frequency-of-occurrence” question. The responses to the frequency-of-occurrence question included rarely (once or twice) coded as 1, sometimes (three to ten times) coded as 2, and often (more than ten times) coded as 3. The HFIAS is generated by summing the frequency-of-occurrence values, resulting in a score ranging between 0 (if household has experienced none of the conditions) and 27 (if household experienced all conditions each more than ten times in the past 4 weeks) [48] Again, due to higher occurrences of severe insecure food access among sample households (48.77%), as presented using online supporting materials (S1 Table), the level HFIAS is also used for analysis.

### 3.4 Empirical model specification

We employ inverse-probability-weighted regression adjustment (IPWRA) to identify the impacts of participation in one of the treatment arms on food security outcomes. The IPWRA has several advantages over other treatment estimators. First, IPWRA is a doubly robust treatment estimator [49, 50] and allows the estimation of treatment assignment and outcome equations in the same framework so that consistent treatment effects can be calculated if either the treatment or the outcome models are correctly specified [51, 52]. The effects of potential misspecification in either propensity score or regression adjustment model can be corrected by combining the propensity score method with regression adjustments [52]. Second, IPWRA estimation provides a choice of functional forms that guarantee the estimated conditional probability and conditional mean functions produce predictions within the logical range of the outcomes [49]. Thirdly, unlike Inverse-probability weighted (IPW) and Propensity-score matching (PSM), the IPWRA has also the outcome model to control for the baseline characteristics associated with the outcome which may not be controlled to predict treatment status. Controlling for other covariates in addition to the treatment status absorbs variance and improves the precision of estimates [20]. Lastly, IPWRA also improves statistical precision as it compares every treatment unit to every other comparison unit while attaching higher weights to those that have a similar likelihood of being in the treatment or comparison group and lower weights to dissimilar observations [53].

Following Hirano and Imbens [54], the ATE using the IPWRA approach is specified as:

$$Y_{i}=\beta_{0}+\tau T_{i}+\beta_{1}Z_{i}+\beta_{2}\left(Z_{i}-\bar{Z}\right)T_{i}+\varepsilon_{i}$$

where *Y*_*i*_ is the outcome variable (food security indicators), *T*_*i*_ is the treatment indicator, *Zi* is the vector of covariates in the outcome equation, $\bar{Z\;}$ is the sample average of *Z* for the sub-sample of the households that participated in the programs, and *ε*_*i*_ is the error term. The weights are given as:

$$\omega \left(t,x\right)=\frac{t}{\hat{p}\left(x\right)}-\frac{1-t}{1-\hat{p}\left(x\right)}$$

where, *ω(t*,*x)* is the weight, *t* represents *Ti = 1*, *x* denotes a vector of covariates in the propensity score equation, and $\hat{p}\left(x\right)$ represents the estimated propensity score. Accordingly, using predicted outcomes of treatment and control households, the ATE is calculated as follows:

$$ATE=E\left[{\hat{Y}}_{i}|T_{i}=1\right]-E\left[{\hat{Y}}_{i}|T_{i}=0\right]$$

The propensity score is calculated from the probability of being treated and given by *Pr(Ti) = f(X)*, where *Pr(Ti)* is the probability of participation in one of the three treatment arms and X is a vector of covariates. Again, the ATT can be expressed as:

$$\begin{array}{ll} ATT_{IPWRA} & =\;\frac{1}{n_{T}}\sum_{i=1}^{n_{T}}T_{i}\left[r_{T}^{*}\left(X,\gamma_{T}^{*}\right)-r_{C}^{*}\left(X,\gamma_{C}^{*}\right)\right]\\ & =\;\frac{1}{n_{T}}\sum_{i=1}^{n_{T}}\left[\left(\hat{\delta_{T}^{*}}-\hat{\delta_{C}^{*}}\right)-\left(\hat{\varphi_{T}^{*}}-\hat{\varphi_{C}^{*}}\right)X_{i}\right] \end{array}$$

where n_T_ is the number of CCT beneficiary and CBHI insured, CCT beneficiary, or CBHI insured households (T) and *r*_*i*_*(X)* is the regression model for the treated households and control (C) households based on observed covariates *X* and parameters *γ*_*i*_ *= (δ*_*i*_, *φ*_*i*_*)*.

where $\gamma_{T}^{*}=\left(\delta_{T}^{*}{,\varphi }_{T}^{*}\right)$ (inverse probability weighted parameters for treated households) is obtained from a weighted regression procedure of:

$$\underset{\delta_{T,}^{*}\varphi_{T}^{*}}{\text{min}}\sum_{i=1}^{N}T_{i}{\left(Y_{i}-\delta_{T}^{*}-X\varphi_{T}^{*}\right)}^{2}/\hat{p}\left(X,\hat{\beta }\right)$$

and $\gamma_{C}^{*}=\left(\delta_{C}^{*}{,\varphi }_{C}^{*}\right)$ (inverse probability weighted parameters for control households) is obtained from a weighted regression procedure of:

$$\underset{\delta_{C,}^{*}\varphi_{C}^{*}}{\text{min}}\sum_{i=1}^{N}{\left(1-T\right)}_{i}{\left(Y_{i}-\delta_{C}^{*}-X\varphi_{C}^{*}\right)}^{2}/\left(1-\hat{p}\left(X,\hat{\beta }\right)\right)$$

We used Stata’s Treatment Effects command to estimate both ATE and ATT.

The IPWRA estimators require several assumptions including the conditional independence assumption (CIA) or unconfoundedness, independent and identically distributed (IID) observations, and overlap to satisfy. The CIA states that the potential outcomes are independent of treatment assignment once we control for all observable baseline variables. However, this is a strong assumption as self-selection into treatment might still be based on unobservables [52]. The IID requires that the potential outcomes and treatment status of each individual are independent of the potential outcomes and treatment status of all other individuals in the population. Finally, the overlap assumption states that each individual in the sample has a positive estimated probability of receiving treatment [55].

### 3.5 Covariate selection

Covariates selected for the model include those related to the head such as sex and education status and household-related factors including ownership of farmland and livestock, household size, household’s participation in other food security-related interventions and agricultural training, access to credit, income from non-PSNP employment sources, and membership in a community or local associations and groups. We also controlled for village characteristics such as walking distances in minutes from home to the nearest health center and agroecological zone of the villages. These variables are assumed to be less affected by the household’s inclusion into PW (CCT) and CBHI enrolment to meet the ignorability assumption. The selection of the variables in the estimation of the probability of membership in CBHI and inclusion in CCT was guided by theory and similar past studies on enrollment in CBHI [56–59] and food security [60, 61]. Further, based on the program implementation manual [15], the contextual factors related to targeting the CCT were also controlled.

## 4. Results

### 4.1 Descriptive results

Table 1 presents the demographic, socioeconomic, and village characteristics of households according to their membership in CCT and CBHI. For the full sample, the average age of the heads of households is 40.29 years, and 35% of heads are literate. We also find that study households have an average of 5.12 people, 32% of households own livestock, and only 28% have agricultural land. This figure supports the fact that PSNP beneficiary households are poorer and have a small size or no farmlands and livestock. Participation in other food security programs (OFSP) is 16% among the pooled sample, and membership in local associations and groups is 21%.

**Table 1:** Sample characteristics.

	(1)		(2)		(3)		(4)	
	Pooled		Insured & CCT		Insured		CCT	
	Mean	SD	Mean	SD	Mean	SD	Mean	SD
*Household characterizes*								
Age of female head	40.29	10.15	41.96	9.82	40.90	9.23	42.01	10.16
The female household head is literate (1/0)	0.35	0.48	0.63	0.49	0.51	0.50	0.54	0.50
Household size	5.12	2.13	5.36	2.03	5.30	2.01	5.44	2.22
Household owns livestock (1/0)	0.32	0.47	0.51	0.50	0.47	0.50	0.41	0.49
A household has farmland (1/0)	0.28	0.45	0.40	0.49	0.41	0.49	0.33	0.47
Household participates in one or more OFSP (1/0)	0.16	0.37	0.27	0.44	0.24	0.43	0.21	0.41
Household is a member of any association in the village (1/0)	0.21	0.41	0.35	0.48	0.29	0.45	0.29	0.45
Income from non-PSNP employment (log)	2.43	3.85	1.76	3.51	1.62	3.36	1.94	3.62
Household received agriculture training last 12 months (1/0)	0.23	0.42	0.42	0.49	0.35	0.48	0.33	0.47
Household received credit last 12 months (1/0)	0.35	0.48	0.57	0.50	0.50	0.50	0.49	0.50
*Village characteristics*	Freq.	Percent	Freq.	Percent	Freq.	Percent	Freq.	Percent
Livelihood zones								
*Tekeze* lowland	147	40.27	58	40.85	102	43.97	72	40.45
*Woyna Dega* mixed cereals	117	32.05	51	35.92	76	32.76	57	32.02
*Tana Zuria*	101	27.67	33	23.24	54	23.28	49	27.53
Distance from home to the nearest health center								
Below 15 minutes	97	26.58	49	34.51	77	33.19	53	29.78
15–30 minutes	58	15.89	18	12.68	39	16.81	28	15.73
31–60 minutes	76	20.82	22	15.49	37	15.95	33	18.54
Above 60 minutes	134	36.71	53	37.32	79	34.05	64	35.96
Observations	365		142		232		178	

Note: for dummy covariates (1/0): 1 = yes, 0 = no

Regarding participation in agricultural training and access to credit, we find that 23% and 35% of pooled households have received agriculture training and credit in the last 12 months, respectively. A summary of village characteristics shows that 40.27% of households reside in *Tekeze* lowland, 32.05% in the *Woyna Dega* mixed cereal, and 27.67% in the *Tana Zuria* livelihood zones. What is more, the results also show that 26.58% of sample households are located within 15 minutes, 15.89% between 15 and 30 minutes, 20.82% between 31 and 60 minutes, and 36.71% above 60 minutes walking-distance away from the nearest health center.

### 4.2 Membership in social protection programs

Table 2 presents descriptive statistics about membership in CCT and CBHI. We find that 63.6% of households are enrolled in CBHI and participation in the PSNP’s CCT component is 48.8%. Furthermore, the results show that 38.9% of sample households are members of both CBHI and CCT programs.

**Table 2:** Membership in social protection programs.

	Mean	SD
Percentage of CBHI-insured households	0.636	0.482
Household is PSNP’s CCT beneficiary (1/0)	0.488	0.501
The household is enrolled in CBHI and is a CCT client (1/0)	0.389	0.488
Observations	365	

### 4.3 Food security outcomes

Table 3 presents descriptive statistics on the proportion of households who consumed the 12 food groups in the past 24 hours, followed by the HDDS, HFCS and HFIAS indicators. Results show that while all households consumed cereal foods in the past 24 hours, the next most consumed food groups were any condiments or spices like tea, coffee, salt, garlic, spices, or yeast (87.1%) and any legumes or nut like beans, cowpeas or lentils (75.9%). It is also showed that no household consumed fish in the 24 hours prior to the survey date.

**Table 3:** Descriptive statistics on 12 food items and food security scores.

Food groups and scores	Mean	SD
Any cereals and grain like teff, wheat, sorghum, millet or maize	1.000	0.000
Any Roots and tubers like potato, cassava or sweet potato	0.230	0.421
Vegetables	0.460	0.499
Fruits	0.211	0.409
Meat	0.164	0.371
Any eggs	0.430	0.496
Any fish	0.000	0.000
Any legumes/ nut like beans, cowpeas or lentils	0.759	0.428
Any milk and other dairy products like yogurt or cheese	0.260	0.439
Any oil/ fat/ butter like vegetable oil, palm oil, butter, or margarine	0.636	0.482
Any sugar, or sweet like sugar, honey, candy, cookies, or cakes	0.208	0.407
Any condiments/ spices like tea, coffee, salt, garlic, spices, or yeast	0.871	0.335
Household dietary diversity index (12 food groups)	1.970	0.947
Household food consumption score (9 food groups)	1.890	0.901
Household food insecurity access scale	12.915	9.971
Observations	365	

Table 3 also presents food security outcome indicators. We find that sample households have a dietary diversity score (based on 12 food groups) of 1.97 (extremely low variety by any standards) and a food consumption score of 1.89. Moreover, our sample households are extremely poor and among the most vulnerable groups where they consume 2 food items only. The results clearly show the households’ inability to consume diversified food which could be due to their limited economic ability. On the other hand, the household food insecurity access scale was 12.92. As per this result, the sample households are more likely to have experienced food insecurity (access component) in the past 4 weeks.

### 4.4 Treatment estimations

#### Density plots

**Fig 1:**
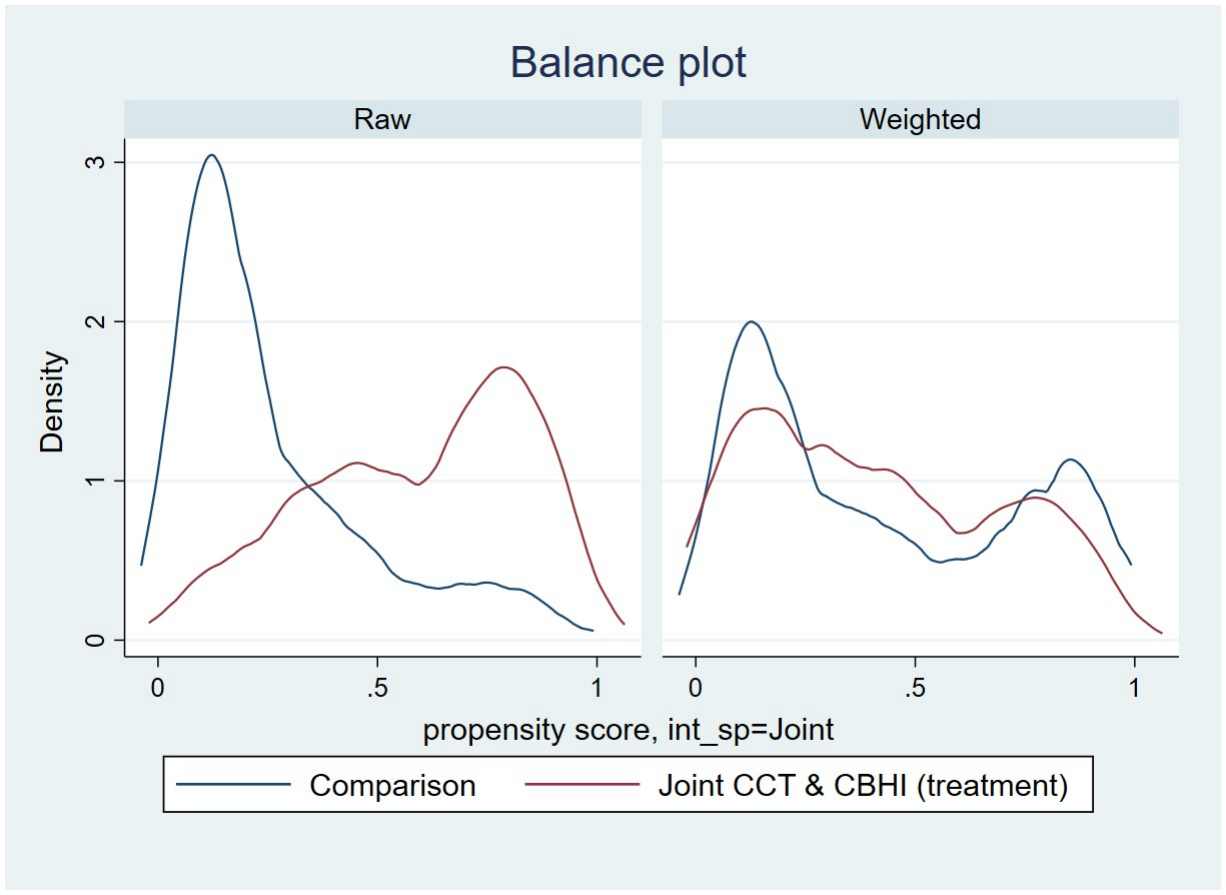
Distribution of propensity scores between treatment arms before and after weighting.

Fig 1 presents the distribution of propensity scores for treatment (members of both CCT and CBHI programs) and comparison households (members of one of the two programs plus none). The density graphs show that propensity scores for comparison and treatment groups were similar after weighting with clear overlap. This shows that sample households are equally likely to be included in both CCT and CBHI programs. We provided the density plots for the membership in CCT and enrolment in CBHI in the online supporting materials (S1 and S2 Figs).

#### Covariate balancing

Using the absolute standardized mean difference of covariates (ASMD) [62] and the ratio between variances [63, 64], we also checked if the balance has been achieved between covariates between comparison and treatment households. These methods are important to create similar groups of households so that differences in the outcomes can be attributed to the inclusion in one or both social protection programs. The conditional independence assumption is said to be fulfilled when covariates are balanced. We used the recommended values of ASMD below 0.1 (< 10%) [63, 65] and the variance ratios close to 1.0 [63, 64] or generally between 0.5 and 2 [64] to achieve balanced covariates.

Table 4 presents a summary of ASMD and variance ratios before and after weighting for the membership both in CBHI and CCT programs. The standardized differences in mean and proportion for weighted covariates are all below 0.1 and variance ratios are mostly close to 1.0 and all are within the acceptable region. The absolute standardized differences range between 0.003 for Tana Zuria livelihood category and 0.096 for residing within 15–30 minutes of walking distance from home to the nearest health center. The variance ratios range between 0.794 for residing 15–30 minutes walking distance from home to the nearest health center and 1.099 for residing 31–60 minutes walking distance from home to the nearest health center. These tests suggest that we achieved a balance in covariates between comparison and treatment groups on these observable variables. Balanced covariates suggest that regression adjustment has adequately eliminated bias due to observable factors when comparing outcomes between treatment arms. The covariate balance summaries for membership in CCT and enrolment in CBHI treatment arms are provided in the online supporting materials (S2 and S3 Tables).

**Table 4:** Covariate balance summary.

	Standardized differences		Variance ratio	
	Raw	Weighted	Raw	Weighted
Age of female head	0.272	-0.022	0.922	0.939
Age squared	0.256	-0.029	1.104	1.038
Household size	0.185	-0.045	0.862	0.937
Head is literate	1.022	-0.046	1.593	0.978
Household owns farmland	0.456	-0.084	1.521	0.931
Household owns livestock	0.724	-0.079	1.638	0.946
Income from non-PSNP employment (log)	-0.292	-0.081	0.771	0.927
Household is a member of any village-level associations	0.577	0.037	2.221	1.053
Household participates in agricultural training in the past 12 months	0.746	-0.081	2.535	0.914
Walking distances from home to the nearest health center in minutes (Ref.: below 15 minutes)				
15–30 minutes	-0.146	-0.096	0.754	0.794
31–60 minutes	-0.219	0.069	0.715	1.099
Above 60 minutes	0.021	-0.051	1.014	0.968
Livelihood zones (Ref.: *Tekeze* lowland)				
*Woyna Dega* mixed cereals	0.135	0.078	1.107	1.052
*Tana Zuria*	-0.164	-0.003	0.844	0.996

#### Overidentification test for covariate balance

we also used the *tebalance overid* command—overidentification test for covariance balance [66], to test whether covariates were balanced. The null hypothesis for the test is ‘covariates are balanced’. Our *chi*^2^ test = 11.28 and *p* = 0.73) showing that we failed to reject the null hypothesis, meaning that both treatment and comparison groups for membership in both programs are balanced on observables. Similarly, we also failed to reject the null hypothesis (H0: Covariates are balanced) for the inclusion in CCT (*chi*^2^ = 13.82 and *P* = 0.54) and enrolment in CBHI (*chi*^2^ = 4.08 and *P* = 0.94).

### 4.5 Impact estimations

Tables 5 and 6 present the treatment effects of participation in the two large-scale social protection programs on food security outcomes. We estimated the impacts of 1) participation in the CCT program, 2) enrollment in CBHI, and 3) participation in both CBHI and CCT programs.

**Table 5:** Treatment effects of social protection programs on food security outcomes: ATE.

	Treatment Effects	Robust Std. Err.	Potential outcomes
**Treatment arms**	**Impacts on HDDS**		
CCT and CBHI	0.918***[0.779,1.057]	0.071	1.675***[1.570,1.781]
CCT	0.748***[0.603,0.893]	0.074	1.670***[1.560,1.780]
CBHI	0.302**[0.055,0.549]	0.173	1.810***[1.584,2.036]
	**Impacts on HFCS**		
CCT and CBHI	0.576***[0.464,0.688]	0.057	1.684***[1.585,1.783]
CCT	0.568***[0.447,0.688]	0.061	1.660***[1.557,1.762]
CBHI	0.271***[0.103,0.440]	0.112	1.692***[1.538,1.847]
	**Impacts on HFIAS**		
CCT and CBHI	-8.658***[-9.775,-7.541]	0.570	15.48***[14.35,16.61]
CCT	-7.575***[-8.861,-6.288]	0.656	15.90***[14.72,17.09]
CBHI	-2.857**[-5.398,-0.316]	1.297	15.08***[12.68,17.49]
Observations	365		

Note: 95% confidence intervals in brackets, significance levels:

* *p* < 0.1,

** *p* < 0.05,

*** *p* < 0.01.

Robust standard errors are reported.

**Table 6:** Treatment effects of social protection programs on food security outcomes: ATET.

Treatment arms	Treatment effects	Robust Std. Err.	Potential outcomes
	**Impacts on HDDS**		
CCT and CBHI	0.712***[0.550,0.874]	0.082	1.943***[1.764,2.122]
CCT	0.609***[0.450,0.769	0.081	1.919***[1.748,2.090]
CBHI	0.243[-0.097,0.582]	0.168	2.072***[1.742,2.401]
	**Impacts on HFCS**		
CCT and CBHI	0.526***[0.392,0.660]	0.069	2.016***[1.857,2.176]
CCT	0.478***[0.351,0.605]	0.065	1.938***[1.791,2.085]
CBHI	0.281**[0.061,0.501]	0.112	1.926***[1.707,2.145]
	**Impacts on HFIAS**		
CCT and CBHI	-6.734***[-8.256,-5.213]	0.776	11.81***[9.810,13.81]
CCT	-7.575***[-8.861,-6.288]	0.831	15.90***[14.72,17.09]
CBHI	-3.374*[-6.908,0.160]	1.823	12.52***[8.948,16.09]
Observations	365		365

Note: 95% confidence intervals in brackets, significance levels:

* *p* < 0.1,

** *p* < 0.05,

*** *p* < 0.01.

Robust standard errors are reported.

### 4.6 Average treatment effects

We employed IPWRA treatment estimates on the full sample of households to estimate the causal effects. The average treatment effects (ATE) (Table 5) show the average amount by which respective food security outcomes are impacted by households’ participation in both programs, enrollment in CBHI, and inclusion in the CCT. Treatment effects on the treated (ATET) (Table 6) present the average amount by which the respective food security outcomes among treatment households are affected due to households’ participation in both programs, enrollment in CBHI, and inclusion in the CCT program. Results show that membership in social protection programs has significant impacts on all food security outcomes. However, the results also revealed that participation in both programs led to larger impacts on food security outcomes compared to participation in one of the programs alone, providing suggestive evidence about the synergy between the two programs.

#### Participation in both programs

Participation in CCT and enrolment in CBHI has a significant impact on all food security outcomes. The ATE shows that participation in CCT and CBHI programs increased household dietary diversity score (HDDS) by an average of 0.918 and household food consumption score (HFCS) by an average of 0.576. It also reduced the household food insecurity access scale (HFIAS) by an average of 8.658.

#### Participation in CCT

Participation in CCT also significantly increased household HDDS and HFCS and reduced HFIAS. We find that participation in CCT increased HDDS by an average of 0.748 from the potential HDDS of households who are not beneficiaries of CCT. However, the effect size is lower than the joint impact of the programs. Participation in CCT also increased HFCS by an average of 0.568 and reduced HFIAS by an average of 7.575. Comparing the effect sizes, it is noted that joint membership has also a larger impact on increasing HFCS and reducing HFIAS compared to participation in the CCT program. For the CCT participants, it should be noted that the comparison households could also be CBHI members.

#### Enrolment in CBHI

Table 5 also shows the impacts of enrolment in CBHI on household HDDS, HFCS, and HFIAS. The results show that enrolment in CBHI also increased household HDDS and HFCS, and reduced HFIAS. The estimate on HDDS suggests that CBHI enrollment increased the HDDS by an average of 0.302 and HFCS by an average of 0.271. The change also shows that the impacts of CBHI on HDDS and HFCS were much lower compared to the joint impacts of the programs. On the other hand, the results show that HFIAS decreased by 2.857 due to households’ enrolment in CBHI.

The ATE and ATET estimates may differ due to the differences in the distribution of covariates between treatment and comparison households and selection biases in treatment assignments. Among the respective treatment households, although the magnitudes vary, we find that all the food security outcomes are significantly affected by membership in both social protection programs and participation in CCT (Table 6). On the contrary, enrolment in CBHI significantly increased HFCS and reduced HFIAS among treatment households, but no statistically significant impact was found related to HDDS. It is also generally observed that the impacts of CBHI alone on food security outcomes among treated households were not as large as impacts of CCT alone and membership in both programs.

### 4.7 Inverse probability weighting estimator

We used an inverse-probability-weighted (IPW) estimator to check the robustness of estimates for participation in both programs and binary logistic regression for the treatment model. The key difference between these two techniques is that while IPWRA directly incorporates the weights into the regression model for the outcome, IPW creates a pseudo comparison group based on covariates used for balancing. In both estimators, we ascertain some robustness based on observed characteristics. The estimates presented in Table 7 show the impacts of joint membership in CCT and CBHI on HDDS, HFCS, and HFIAS. Before generating the estimates, we also conducted balance checks and ensured that the covariates were balanced and there was a significant overlap of propensity scores between treatment and comparison households. Furthermore, the overidentification test for covariate balance shows that covariates are balanced.

**Table 7:** Robustness check for membership in both CCT and CBHI on food security outcomes using IPW estimator.

Food security indicators	ATE	Robust	ATET	Robust
		Std. Err.		Std. Err.
HDDS	0.826***[0.631,1.021]	0.0996	0.660***[0.343,0.977]	0.1619
FCS	0.527***[0.361,0.693]	0.0845	0.449***[0.186,0.712]	0.1343
FIAS	-7.731***[-9.377,-6.085]	0.8397	-5.809***[-8.394,-3.226]	1.3184
Observations	365		365	

**Note:** Significance levels:

* *p* < 0.1,

** *p* < 0.05,

*** *p* < 0.01;

Robust standard errors are reported. All food security measures are made at the household level.

Except for slight reductions in the magnitude of treatment effects, the results were consistent in significance levels and direction for the change in the estimator from IPWRA to IPW. Our results also consistently show that food security outcomes significantly improved, and the household food insecurity access scale reduced among female-headed households due to households’ membership in both social protection programs compared to those who joined either only one of the programs or none.

## 5. Discussion

This study examines the impact of Ethiopia’s largest social protection programs, the conditional cash transfer component of the Productive Safety Net Program (PSNP-CCT component) and the Community-based Health Insurance Program (CBHI), on various food security outcomes among female-headed households in Ebinat district, Amhara region, Ethiopia. The study adds new evidence on 1) the joint impacts of inclusion in the CCT and enrollment in CBHI; 2) membership in the CCT and 3) enrolment in CBHI on HDDS, HFCS, and HFIAS. In this dimension, this study contributes to recent studies that assess the joint impacts of social protection interventions, including Shigute and colleagues [29] who studied effects on labor supply, household assets and financial inclusion in Ethiopia. The study is the first that tries to examine the joint and separate casual impacts of social protection programs on food security outcomes in Ethiopia among female-headed households. The evidence is expected to improve the understanding of how integrating social protection programs could impact food security outcomes among the most vulnerable and marginalized groups in a developing country. We find that membership in CCT and enrolment in CBHI together significantly improved HDDS and HFCS, and reduced HFIAS. However, although we also find that membership in either CCT or CBHI has improved food security outcomes substantially, the impacts were not as large as the joint impacts.

The study finds that by and large, combining CBHI and CCT had far greater effects on household food insecurity than any of CBHI or CCT alone. The results are not small, in either their magnitude or their level of significance. Taking from our preferred estimates from the IPWRA analysis, we observed that a combination of CCT and CBHI led to increasing the number of food groups a female-headed household consumed by about 0.918 food groups, increase household food consumption score by 0.576 and led to 8.658 points decline on household food insecurity access scale. Similarly, CCT alone led to an increase in HDDS of about 0.748 food groups, an increase in HFCS of about 0.568 and a decline in HFIAS of about 7.575 points on the HFIAS scale. CBHI had the least effects on household food insecurity–though they were all statistically significant. We observed that CBHI led to a 0.302 increase in the number of food groups consumed, a 0.271 increase in HFCS score and a 2.857 decline on the HFIAS scale.

Putting the above results in their true magnitudes, we used the non-linear combinations and also extracted the delta-method standard errors and show these results in Table 8 below. We find that the combination of CBHI and CCT programs increases HDDS by 54.8% and HFCS by 34.2% and reduces HFIAS by 54.5%.

**Table 8:** Proportional effects of CBHI and CCT on food insecurity outcomes.

Proportional changes on HDDS						
Outcome	Coefficient	Std. err.	z	P>z	[95% conf. interval]	
CCT & CBHI	54.8%	0.054	10.04	0.000	0.441	0.655
CCT	44.6%	0.054	8.32	0.000	0.341	0.551
CBHI	16.3%	0.079	2.07	0.038	0.009	0.318
Proportional changes on HFCS						
CCT & CBHI	34.2%	0.041	8.43	0.000	0.262	0.421
CCT	34.2%	0.044	7.8	0.000	0.256	0.423
CBHI	16.0%	0.057	2.82	0.005	0.049	0.272
Proportional changes in HFIAS						
CCT & CBHI	-54.5%	0.028	-19.55	0.000	-0.599	-0.491
CCT	-47.0%	0.032	-14.84	0.000	-0.532	-0.408
CBHI	-19.7%	0.052	-3.76	0.000	-0.299	-0.095

Notes: Standard errors are the delta-method standard errors extracted after applying the non-linear combination on the main regressions in Table 5.

In some way, the effect of CCT and the combination of CCT and CBHI was more of less the same on HFCS–about 34.2%. One key postulation we can make about why this is the case is the possibilities of cash and choice in social protection. On the other hand, CBHI increased HDDS and HFCS by 16.3% and 16.0% respectively and reduces HFIAS by 19.7%. Whereas CBHI provides needed protection in times of health shocks, it does not put cash in the hands of households. It therefore can only increase household consumption of food and other goods through its effect on labor markets [29, 67]. On the other hand, CCT puts cash directly in the hands of poor households and therefore household food consumption is immediately positively affected.

By and large, our choice of adopting three different measures of food security is justified by the differences we observe. For instance, by using HFCS only, we might not observe the large effects that accrue on either HDDS or HFIAS. We can also observe that in a case like ours, regarding poor female-headed households, smaller changes in shorter period (7-days) food consumption score can also manifest as larger changes in longer recall measures such as the 30-day HFIAS measure due to the CCT monthly cash transfer. We are therefore more confident that a multiple and multi-dimensional food security measures like these provide us a more comprehensive picture of the effects of combined social protection programs.

Although we find no past empirical studies that examined the joint impacts of CCTs and enrolment in CBHI on food security, previous studies on CT programs support our findings related to household dietary diversity. Existing literature shows that household dietary diversity can be increased through CT programs (conditional and unconditional) in Ethiopia [68] and other countries including Malawi [68, 69], Kenya (cash transfer scheme for orphans and vulnerable children (OVC)) [68, 70], and Uganda [71]. Likewise, in line with our findings concerning food consumption score, cash transfers increased households’ food consumption in Malawi [69, 72], Kenya (unconditional CTs programs) [72–75], Uganda [71], Ethiopia [72], and Zambia [72, 76]. Furthermore, past studies [77, 78] also reported similar results related to the household food insecurity access scale. In Malawi, Abdoulayi et al. [78] find that the social CT scheme increased the number of meals eaten per day. Handa et al. [77] also find that Ghana’s LEAP reduced negative coping strategies such as eating less food and cutting the number of daily meals in case of food insecurity which are some of the indicators of household food insecurity access scale. However, our results contrast with some past studies such as Merttens et al. [75] who did not find impacts of Kenya’s HSNP on increasing food security outcomes. Handa et al. [77] also reported that LEAP had no significant impact on household food consumption.

Our findings have useful policy implications, particularly to realize sustainable development goals related to hunger and food security (Goal 2. End hunger, achieve food security, and improved nutrition and promote sustainable agriculture) and universal health coverage (Goal 3. Ensure healthy lives and promote wellbeing for all at all age) among vulnerable groups. In 2015, the UN member states pledged to leave no one behind and committed to “end hunger and ensure access by all people, in particular, the poor and people in vulnerable situations, including infants, to safe, nutritious and sufficient food all year round” (Target 2.1) by 2030 [79].

Although Ethiopia’s performance in achieving zero hunger (Goal 2) and good health and well-being (Goal 3) show moderately improving trends, gaps remain in reaching the goals by 2030 [80]. For instance, Ethiopia had 44.2 scores in the universal health coverage tracer index (0–100) in 2017 [79] and only 12.5% (125 people out of 1000) of the population has health insurance coverage in 2016/17 [81]. In this regard, although CBHI has the potential to improve the health status of vulnerable households, thereby increasing their food security outcomes due to lower financial expenses on health services and better income, CBHI enrolment is not universal (64% in our sample). This calls for policy measures to increase their enrolment such as through partial or full premium fee waiver by the government or implementing a cross-subsidizing health insurance system.

In addition to food security, the integration of social protection programs may also have synergy effects to achieve other socioeconomic outcomes. As the CCT could alleviate some of the households’ financial constraints that limit households’ capability and enrolment in CBHI reduces the risks of catastrophic health expenditure, households may allocate their incomes to access other essential social services such as education and reduce riskier behaviors and practices induced by poverty such as child marriage. The national social protection policy (NSPP) also recognizes the role of integrating social protection programs to effectively protect poor and vulnerable households from the multiple adverse effects of shocks and destitution. The policy underscores that the integration of programs improves access to equitable and quality health services and could accelerate the social transformation process by meeting the needs of the most vulnerable households [82]. Inclusion in CCT and enrolment in CBHI among the most vulnerable groups such as female-headed households also prevent the risk of resorting to negative coping strategies such as reducing food intake and distress productive asset sales in a time of shocks.

Furthermore, removing female-headed households’ financial barriers to access essential social services through cash transfers and enrolment in CBHI is an important step to making economic development more inclusive and an innovative strategy to realize gender equality by 2030. However, it should be noted that sample households have lower bases in all the food security indicators considered in the study. This is expected as households should be chronically food insecure and most vulnerable to extreme poverty to be targeted by PSNP. This situation suggests that more concerted efforts are also required to achieve the SDG goals among female-headed households.

Our study has also relevant policy implications to facilitate the graduation of beneficiaries. The PSNP envisioned that through a combination of regular and predictable cash and/or food transfers over at least 3 years and a range of household and environmental asset-building supports, households would build their asset base and become more resilient and self-reliant. However, past empirical studies showed that the PSNP underperformed on these fronts, leading to pre-mature graduation [83]. For example, in 2014, a study found that 19% of recent graduates had a two-month food gap, and 24% had a food gap of three months or more [83]. Furthermore, data from various districts showed that between September 2013 and July 2014, the median district graduated only one in every four PSNP beneficiaries [83]. In this regard, previous studies underscored the role of linking social protection programs for graduation and to sustainability increase food security [84]. Concerning this, our study suggests that integrating the PSNP with other social protection programs such as CBHI may increase the program’s impacts on food security, accelerate graduation and reduce vulnerability to other shocks such as high out-of-pocket health expenditure which could reverse the gains. Devereux [84] also argues that a single instrument cannot achieve food security, and emphasized the need to integrate social protection packages to sustainably achieve food security and graduate households from dependence on social protection programs. In doing so, efforts must be made to ensure social protection programs are designed and implemented in an integrated manner. The NSPP also aims to link existing programs to maximize synergies and accelerate the graduation process [82].

Finally, although our study showed the great potential of integrating CCT and CBHI programs for enhanced food security outcomes, policy documents show that institutional frameworks, capacities, and operational mechanisms are not yet sufficiently strong to drive synergies and maximize impacts for poor and most vulnerable people [82]. The NSPP also clearly stated that the integration of social protection programs needs a harmonized targeting process with a centralized information system and alignment of the targeting criteria to ensure that households receive a comprehensive and complementary set of services [82].

## 6. Conclusion

This study investigates the impacts of Ethiopia’s flagship social protection program, the PSNP, and CBHI, a voluntary and risk pooling scheme in the informal sector, on household food security outcomes in Ebinat district, Ethiopia. Cross-sectional data were generated by interviewing 365 female-headed households.

Results show that membership in both CCT and CBHI programs has significant impacts on dietary diversity, food consumption, and food insecurity access scale. These food security outcomes have also significantly improved due to participation in CCT and enrolment in CBHI, although the impact sizes were not as large as the joint impacts.

The study gives evidence of how integrating social protection programs could affect food security conditions among poor and chronically food-insecure female-headed households. The findings also highlight the potential of social protection programs, particularly when integrated, to achieve sustainable development goals related to ending hunger and achieving food security (Goal 2) among vulnerable households. This could be one strategy to achieve inclusive economic development and leave no one behind by 2030.

Integrating social protection programs requires some institutional restructuring and collaboration. In this regard, the programs need to jointly redefine the minimum benefit level/ cash transfer, harmonize their selection criteria and process for targeting and retargeting, establish a single registry system for coordination and identification, and coordinate transfers.

Finally, we conclude by highlighting on some limitations of the paper. These should guide the reader in interpreting and inferring from these results. First, the paper uses inverse probability weighting regression adjustment to address the observable difference between treatment and comparison households. While the method is efficient in addressing these differences, the method cannot account for unobserved differences and therefore clean causal estimates are not estimated. Moreover, our analysis is also based on one round of cross-sectional data which does not give us opportunity to observe transitions between different states for the same households. Future work is required especially that which applies more robust methods of causal analysis such as experimental and quasi-experimental designs. In addition, the paper draws from only one region and one district in Ethiopia. This implies that these results, while they have policy relevance across the whole country and other low-income countries at large, they need to be taken in consideration that our study area (Ebinat district) might have peculiar differences that make very different from the rest of the country. Future studies should explore the issue using country-level representative samples to get results that can apply beyond a single region.

## Supporting information

S1 Fig Density plots: Participation in CCT program. (TIF)

S2 Fig Density plots: Enrollment in CBHI. (TIF)

S1 Table Household food insecurity category based on HFIAS. (DOCX)

S2 Table Covariate balance summary: Participation in CCT program. (DOCX)

S3 Table Covariate balance summary: Enrolment in CBHI. (DOCX)

S1 Data (DTA)
