# Supplementary figures and images for: Combining social protection interventions for better food security: Evidence from female-headed households in Amhara region, Ethiopia
Source: PLoS One. 2024 Feb 26;19(2):e0283812. doi: 10.1371/journal.pone.0283812 (PMC10896536; doi:10.1371/journal.pone.0283812)

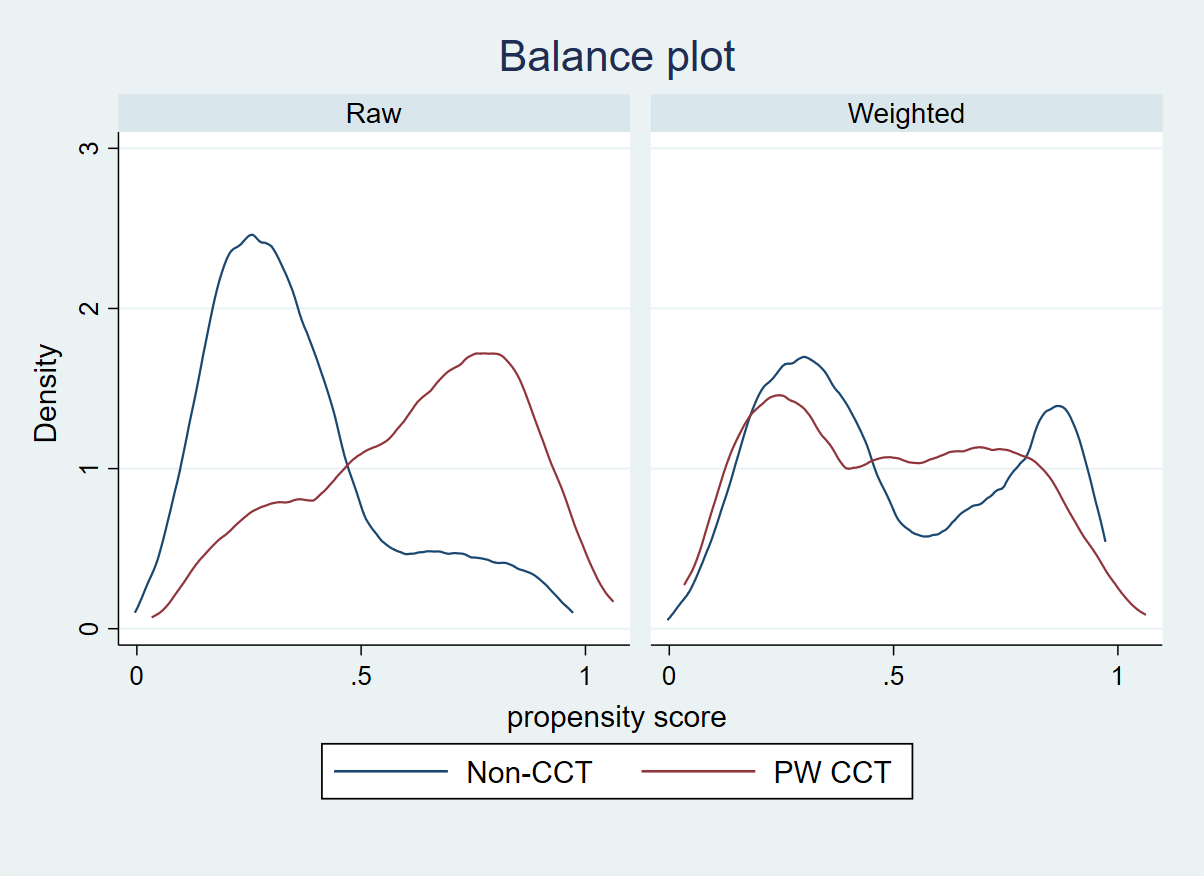

Supplement: S1 Fig — (TIF) [file pone.0283812.s001.tif]

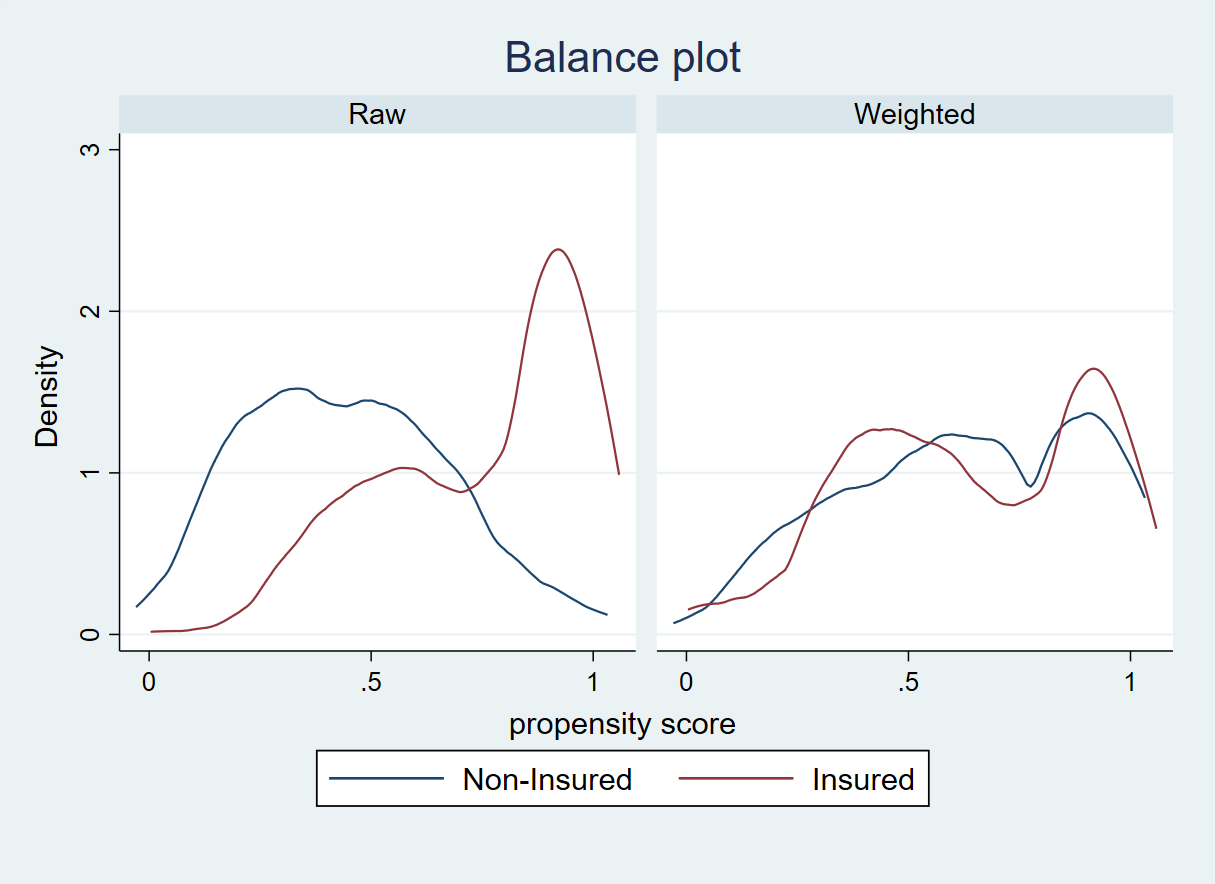

Supplement: S2 Fig — (TIF) [file pone.0283812.s002.tif]
