# Supplementary material for: Combining social protection interventions for better food security: Evidence from female-headed households in Amhara region, Ethiopia
Source: PLoS One. 2024 Feb 26;19(2):e0283812. doi: 10.1371/journal.pone.0283812 (PMC10896536; doi:10.1371/journal.pone.0283812)
Supplement: S1 Table — (DOCX) [file pone.0283812.s003.docx]

**Table 1. Household food insecurity category based on HFIAS**

|  | Frequency | Percent |
| --- | --- | --- |
| Food Secure | 115 | 31.51 |
| Mildly Food Insecure | 22 | 6.03 |
| Moderately Food Insecure | 50 | 13.7 |
| Severely Food Insecure | 178 | 48.77 |
| Total | 365 | 100 |
