# Supplementary material for: Combining social protection interventions for better food security: Evidence from female-headed households in Amhara region, Ethiopia
Source: PLoS One. 2024 Feb 26;19(2):e0283812. doi: 10.1371/journal.pone.0283812 (PMC10896536; doi:10.1371/journal.pone.0283812)
Supplement: S2 Table — (DOCX) [file pone.0283812.s004.docx]

**Table 2. Covariate balance summary: Participation in CCT program**

|  | Standardized differences | | Variance ratio | |
| --- | --- | --- | --- | --- |
|  | Raw | Weighted | Raw | Weighted |
| Age of female head | 0.335 | 0.058 | 1.058 | 1.010 |
| Age squared | 0.335 | 0.059 | 1.235 | 1.095 |
| Household size | 0.298 | 0.036 | 1.228 | 1.153 |
| Head is literate | 0.844 | -0.060 | 1.749 | 0.969 |
| Household owns farmland | 0.215 | -0.014 | 1.241 | 0.986 |
| Household owns livestock | 0.406 | -0.032 | 1.389 | 0.974 |
| Household is a member of any village level associations | 0.381 | -0.043 | 1.766 | 0.944 |
| Income from non-PSNP employment (log) | -0.253 | 0.018 | 0.821 | 1.062 |
| Household participates in agricultural trainings in past 12 months | 0.496 | -0.006 | 1.981 | 0.993 |
| Walking distances from home to the nearest health center in minutes (Ref.: below 15 minutes) |  |  |  |  |
| 15-30 minutes | -0.009 | 0.043 | 0.984 | 1.098 |
| 31-60 minutes | -0.110 | 0.035 | 0.853 | 1.051 |
| Above 60 minutes | -0.031 | -0.011 | 0.983 | 0.994 |
| Livelihood zones (Ref.: *Tekeze* lowland) |  |  |  |  |
| *Woyna* *Dega* mixed cereals | -0.001 | 0.067 | 0.999 | 1.046 |
| *Tana* *Zuria* | -0.006 | 0.081 | 0.994 | 1.110 |
