# Supplementary material for: Combining social protection interventions for better food security: Evidence from female-headed households in Amhara region, Ethiopia
Source: PLoS One. 2024 Feb 26;19(2):e0283812. doi: 10.1371/journal.pone.0283812 (PMC10896536; doi:10.1371/journal.pone.0283812)
Supplement: S3 Table — (DOCX) [file pone.0283812.s005.docx]

**Table 3. Covariate balance summary: Enrolment in CBHI**

|  | Standardized differences | | Variance ratio | |
| --- | --- | --- | --- | --- |
|  | Raw | Weighted | Raw | Weighted |
| Age of female head | 0.160 | 0.038 | 0.640 | 1.024 |
| Age squared | 0.102 | 0.039 | 0.720 | 1.048 |
| Head is literate | 1.093 | 0.050 | 3.581 | 1.034 |
| Number of children aged 18 years and below | 0.437 | 0.044 | 1.542 | 1.662 |
| Number of adults aged 19-64 years | -0.075 | -0.100 | 0.891 | 0.849 |
| Number of elderlies aged 65 years and above | -0.257 | -0.029 | 0.550 | 0.757 |
| Income from non-PSNP employment (Log) | -0.588 | 0.034 | 0.635 | 1.034 |
| Walking distances from home to the nearest health center in minutes | -0.156 | -0.062 | 1.343 | 1.270 |
| Walking distances from home to the nearest drinking water source | -0.163 | -0.057 | 1.325 | 1.342 |
